# Identification of ULK1 as a novel mitophagy-related gene in diabetic nephropathy

**DOI:** 10.3389/fendo.2022.1079465

**Published:** 2023-01-18

**Authors:** Yuan-Yuan Yang, Zhong-Xiuzi Gao, Zi-Hui Mao, Dong-Wei Liu, Zhang-Suo Liu, Peng Wu

**Affiliations:** ^1^ Traditional Chinese Medicine Integrated Department of Nephrology, The First Affiliated Hospital of Zhengzhou University, Zhengzhou, China; ^2^ Institute of Nephrology, Zhengzhou University, Zhengzhou, China; ^3^ Henan Province Research Center for Kidney Disease, Zhengzhou, China; ^4^ Key Laboratory of Precision Diagnosis and Treatment for Chronic Kidney Disease in Henan Province, Zhengzhou, China

**Keywords:** mitophagy, diabetic nephropathy, ULK1, biomarker, mitophagy-related genes

## Abstract

**Background:**

Accumulating evidence indicates that mitophagy is crucial for the development of diabetic nephropathy (DN). However, little is known about the key genes involved. The present study is to identify the potential mitophagy-related genes (MRGs) in DN.

**Methods:**

Five datasets were obtained from the Gene Expression Omnibus (GEO) database and were split into the training and validation set. Then the differentially expressed MRGs were screened and further analyzed for GO and KEGG enrichment. Next, three algorithms (SVM-RFE, LASSO and RF) were used to identify hub genes. The ROC curves were plotted based on the hub genes. We then used the CIBERSORT algorithm to assess the infiltration of 22 types of immune cells and explore the correlation between hub genes and immune cells. Finally, the Nephroseq V5 tool was used to analyze the correlation between hub genes and GFR in DN patients.

**Results:**

Compared with the tubulointerstitium, the expression of MRGs was more noticeably varied in the glomeruli. Twelve DE-MRGs were identified in glomerular samples, of which 11 genes were down-regulated and only *MFN1* was up-regulated. GO and KEGG analysis indicated that several enrichment terms were associated with changes in autophagy. Three genes (*MFN1*, *ULK1* and *PARK2*) were finally determined as potential hub genes by three algorithms. In the training set, the AUROC of *MFN1*, *ULK1* and *PARK2* were 0.839, 0.906 and 0.842. However, the results of the validation set demonstrated that *MFN1* and *PARK2* had no significant difference in distinguishing DN samples from healthy controls, while the AUROC of *ULK1* was 0.894. Immune infiltration analysis using CIBERSORT showed that *ULK1* was positively related to neutrophils, whereas negatively related to M1 and M2 macrophages. Finally, *ULK1* was positively correlated with GFR in Nephroseq database.

**Conclusions:**

*ULK1* is a potential biomarker for DN and may influence the development of diabetic nephropathy by regulating mitophagy.

## 1 Introduction

Diabetic nephropathy (DN) is one of the most common diabetic microvascular complications, contributing to considerable mortality and expense throughout the world (1, 2). DN is characterized by persistent albuminuria or proteinuria, reduced glomerular filtration rate (GFR), and a wide range of pathological changes in the kidney (3). The pathogenesis of DN is complicated and multifactorial, involving many pathways and mediators (4). Recently, selective autophagy induced by autophagy substrate as a trigger has received extensive attention. As one of the extensive-studied sorts of selective autophagy, mitophagy performs a pivotal function in retaining mitochondrial functional and genetic integrity (5), and has been viewed as a key component in the development of DN.

Mitophagy targets damaged and depolarized mitochondria and regulates mitochondrial quality control. The key pathway involved in the initiation of mitophagy is the AMPK/mTOR pathway, which is the transition from anabolism to catabolism in cells. As a conserved substrate of AMPK, ULK1 is essential for autophagy (6). In mammals, the lack of AMPK or ULK1 leads to abnormal accumulation of p62 and mitophagy defects. The PINK1-Parkin pathway is a well-studied mitophagy mechanism for the elimination of dysfunctional mitochondria (7). By phosphorylating Parkin, PINK1 facilitates Parkin’s translocation from the cytoplasm to mitochondria for mitophagy (8). Mutations in *PINK1* and Parkin cause early onset forms of Parkinson’s disease (9, 10). In addition, there are several protein receptor-mediated pathways that related to mitophagy, including FUN14 domain-containing protein1 (FUNDC1), mitochondrial protein Nix (also known as BNIP3L) and BCL2 interacting protein 3 (BNIP3) (11). BNIP3/Nix is involved in mitochondrial membrane depolarization-induced hypoxia or mitochondrial autophagy (12). FUNDC1 interacts with LC3 and participates in autophagy *via* the conservative LC3-interacting region domain (13).

Using a transgenic mouse mito-QC for monitoring mitophagy *in vivo*, researchers found that the kidney is one of the most active mitophagy tissues (14). Mounting evidence revealed that mitophagy is strongly related to the progression of DN. For instance, Xiao et al. demonstrated that mitophagy is significantly reduced in diabetic *db/db* mice, and mitochondrial antioxidant (mitoQ) can improve mitochondrial quality control and renal tubular damage in DN by increasing *PINK1*-mediated mitophagy (15). Another study reported that forkhead-box class O1 (*FoxO1*) promotes mitophagy through a PINK1/Parkin pathway, thereby reducing podocyte damage in diabetic nephropathy (16).

Although growing studies reveal that mitochondrial autophagy is related to the pathogenesis of DN, it remains unknown which mitophagy-related genes (MRGs) are necessary for the development of DN. In this study, we performed bioinformatic analysis to identify new biomarkers for DN that may be linked to MRGs.

## 2 Materials and methods

### 2.1 Collection and preprocessing of data

**Figure 1:**
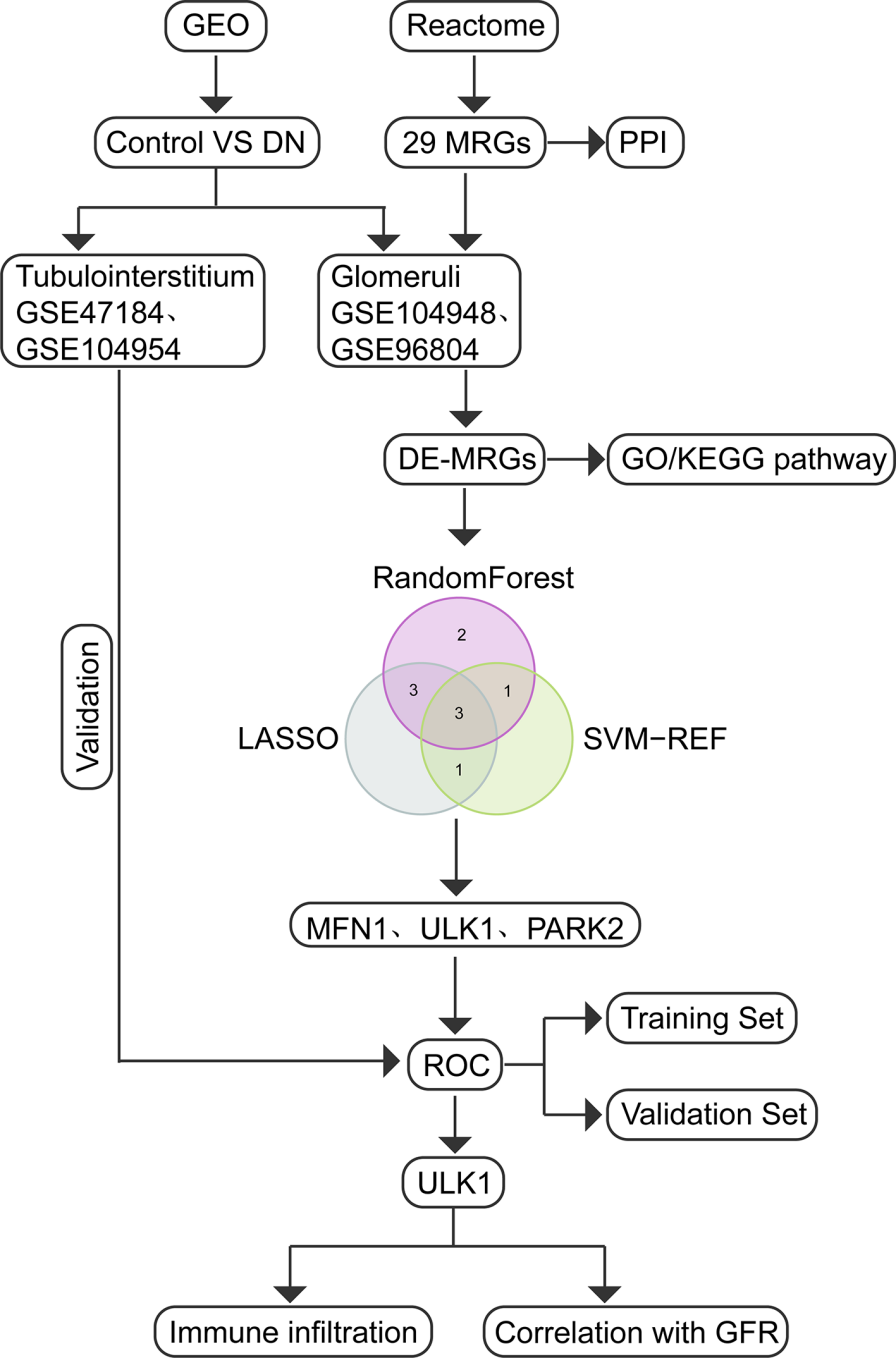
Study flow diagram.

The study flow diagram is presented in **Figure 1**. Five gene expression datasets had been acquired from the Gene Expression Omnibus (GEO) database (17): GSE104948 (18), GSE96804 (19, 20), GSE47184 (21), GSE104954 (18) and GSE30528 (22). Detailed information about the five datasets were showed in **Table 1**. In total, 108 glomeruli samples (57 DN, 51 living healthy donors) and 40 tubulointerstitium samples (18 DN, 22 living healthy donors) were included in our study to evaluate the expression level of MRGs. Gene symbols matched the array probes according to annotation data. A maximum expression value was used to represent the average expression level when mutiple probes corresponded to the identical gene. We used the robust multi-array average expression measure to normalize the data (23). Then we merged different datasets together. The effect of inter-sample correction was demonstrated using a two-dimensional PCA cluster plot. The ComBat function of the R package “sva” (24) was used to remove batch effects, which is the normalization of PCA cluster graph.

**Table 1:** Detailed information about the collected datasets.

Data Type	GEO Series	Platform	Normal	DN	Tissue
Training Set	GSE104948	GPL22945	18	7	Glomerulus
Training Set	GSE96804	GPL17586	20	41	Glomerulus
Training Set	GSE47184	GPL14663	4	11	Tubulointerstitium
Training Set	GSE104954	GPL22945	18	7	Tubulointerstitium
Validation Set	GSE30528	GPL571	13	9	Glomerulus

### 2.2 Identification of differential expressed mitophagy-related genes

Twenty-nine MRGs were extracted from Reactome Pathway Database (https://reactome.org) (**Table 2**). We used the limma package (25) to identify the differentially expressed mitophagy-related genes (DE-MRGs) between the DN patients and controls, and the “heatmap” and “ggplot2” packages were used to draw volcano plot, heatmaps, and box plots. We adjust *P* values by using Benjamini-Hochberg-based False Discovery Rate method. Genes with an adjusted *P*-value <0.05 were considered as DE-MRGs.

**Table 2:** 29 mitophagy-related genes extracted from Reactome Pathway Database.

ATG12	ATG5	CSNK2A1	CSNK2A2	CSNK2B
*FUNDC1*	*MAP1LC3A*	*MAP1LC3B*	*MFN1*	*MFN2*
*MTERF3*	*PGAM5*	*PINK1*	*PARK2*	*RPS27A*
*SQSTM1*	*SRC*	*TOMM20*	*TOMM22*	*TOMM40*
*TOMM5*	*TOMM6*	*TOMM7*	*TOMM70A*	*UBA52*
*UBB*	*UBC*	*ULK1*	*VDAC1*	

### 2.3 Functional enrichment analysis of DE-MRGs

Gene Ontology (GO) and Kyoto Encyclopedia of Genes and Genomes (KEGG) were used to analyze DE-MRGs using the “clusterProfiler” package (26). The GO analysis identified three categories, consisting of biological process (BP), cellular component (CC), and molecular function (MF) (27). Analysis of possible pathways was conducted using KEGG (28).

### 2.4 Identification of optimal hub genes for DN

A support vector machine-recursive feature elimination (SVM-RFE) model was compared by the average misjudgement rates of their 10-fold cross-validations using the “e1071” software package (29). As a novel machine learning technique, SVM-RFE can rank features based on recursion to avoid overfitting (30). As a dimension reduction approach, least absolute shrinkage and selection operator (LASSO) regression exhibits superior performance when evaluating high-dimensional data compared to regression analysis and uses regularization to improve prediction accuracy. A 10-fold cross-verification of LASSO analysis was performed using the “glmnet” package by a turning or penalty parameter (31). Random forest (RF) is a supervised machine learning algorithm built with a decision tree algorithm and is used to solve regression and classification problems. Feature importance was determined by the Mean Decrease Gini Index calculated by RF (32). Using the above algorithms, hub genes for DN were identified. We established the receiver operating characteristic (ROC) curve and the pROC package in R was used to assess the diagnostic significance of mitophagy-related hub genes (33). The area under the ROC curve (AUROC) represented the size of the diagnostic efficiency.

### 2.5 Evaluation of immune cell infiltration

The CIBERSORT tool was used to explore differences in the proportions of 22 types of immunocytes between DN and controls (34). The correlation heatmap was drawn by the corrplot package. Principal component analysis (PCA) clustering and violin diagram of immune cells were performed using the “ggplot2” package.

### 2.6 Clinical correlation between hub genes and renal function in DN patients

We used the Nephroseq V5 tool (https://nephroseq.org/) to evaluate the relationship between hub genes and renal function of DN patients. Then we drawed the scatter plots by the “ggplot2” package.

### 2.7 Statistical analysis

R software was used for all statistical data analysis. Data are the mean value ± standard deviation (SD). Student’s *t*-tests or Wilcoxon tests were used to compare two groups. Three or more groups were compared by the Kruskal-Wallis test. Correlation analysis was assessed using Pearson correlation. *p*-values < 0.05 had been considered statistically significant.

## 3 Results

### 3.1 Landscape of MRGs in DN

**Figure 2:**
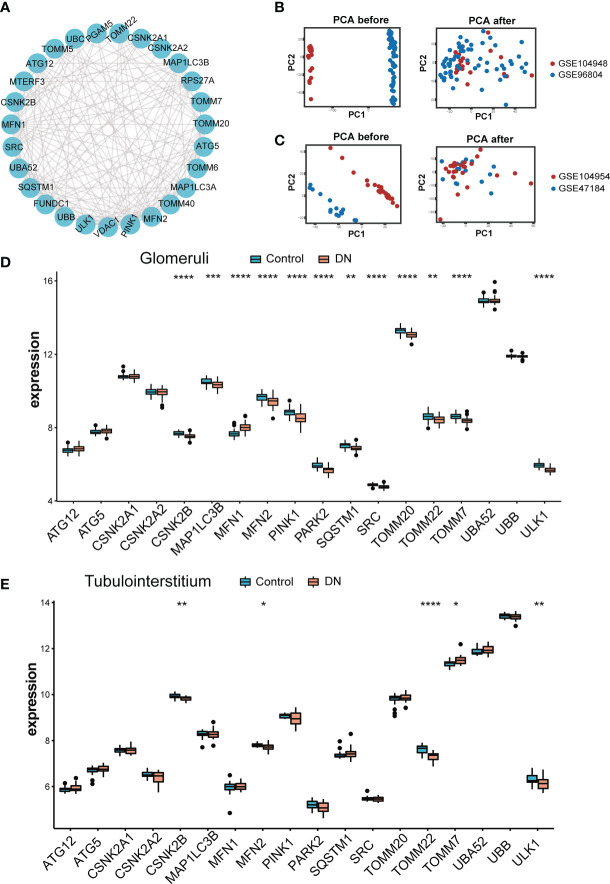
Landscape of mitophagy-related genes in DN. **(A)** Protein-protein interaction (PPI) network made up of 29 mitophagy-related genes. **(B)** The PCA plot of the GSE104948 and GSE96804 datasets before and after sample correction in glomeruli samples. **(C)** The PCA plot of the GSE104954 and GSE47184 datasets before and after sample correction in tubulointerstitium samples. **(D)** Box plot demonstrating the expression level of 18 mitophagy-related genes in glomeruli of living donor and DN. **(E)** Box plot demonstrating the expression level of 18 mitophagy-related genes in tubulointerstitium of living donor and DN. Differences between groups are indicated by “*”. *p < 0.05; **p < 0.01; ***p < 0.001;****p < 0.0001. Data were analyzed by Wilcoxon tests.

The role of twenty-nine MRGs has been currently studied extensively and their interaction was shown in **Figure 2A**. Next, we compared the expression of these MRGs between glomeruli and tubulointerstitium samples. The PCA cluster diagrams in **Figures 2B, C** showed that the clustering of the two samples groups is more obvious after normalization, indicating that the source of the samples is reliable. **Figure 2D** indicated that 18 MRGs were identified in the glomeruli samples and 12 genes showed marked differential expression between DN and living healthy controls (*p* < 0.05), including *CSNK2B*, *MFN1*, *MFN2*, *PINK1*, *PAPK2*, *SRC*, *TOMM20*, *TOMM7* and *ULK1*(*p* < 0.0001). However, as shown in **Figure 2E**, only 5 of a total 18 MRGs identified in tubulointerstitium samples displayed difference in expression. In summary, the most dramatic difference was observed in glomeruli samples. Therefore, glomeruli samples were selected for further study.

**Figure 3:**
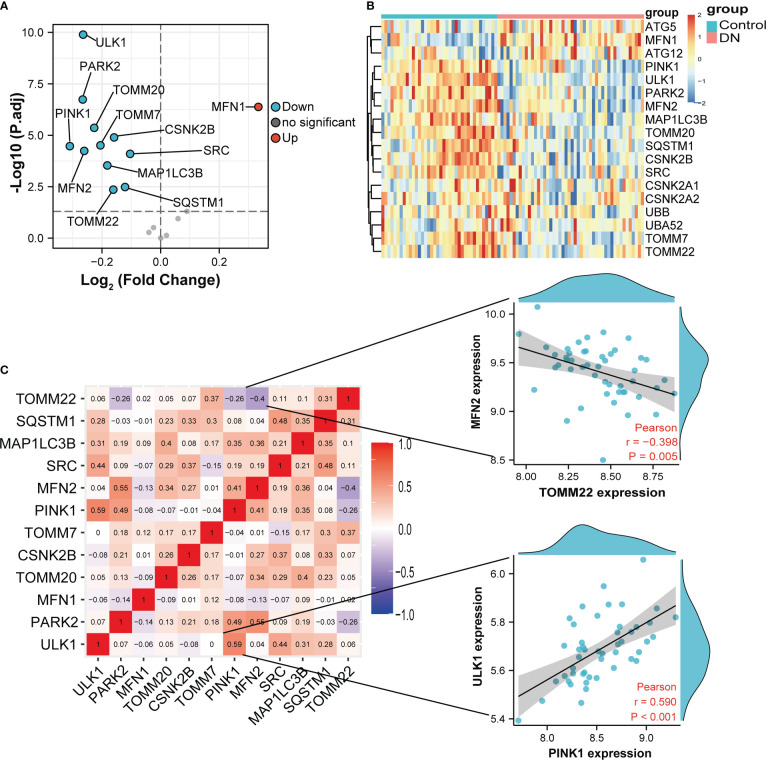
Variance analysis of mitophagy-related genes in DN. **(A)** Volcano plot showing a summary of the expression differences of 18 mitophagy-related genes between the healthy and DN patients’ glomerular samples. **(B)** The clustering heatmap exhibited the expression pattern of DE-MRGs among glomeruli samples. **(C)** Correlations between DE-MRGs in DN glomeruli samples and the respective scatterplots show the two pairs of MRGs with the highest correlation. Correlation analysis was assessed using Pearson correlation.

Volcano in **Figure 3A** showed 12 DE-MRGs, but only *MFN1* was up-regulated and all other genes were downregulated in glomeruli samples. *PINK1* exhibited the greatest fold-change of 0.31 among these downregulated genes, whereas the reduction of *ULK1* was the most statistically significant. The heatmap in **Figure 3B** exhibited the expression of DE-MRGs among glomeruli samples. In correlation analysis (**Figure 3C**), we found that these genes are closely related, suggesting they may function together. The genes with the highest positive and negative correlation were displayed in scatterplots of **Figure 3C**. *MFN2* and *TOMM22* appeared to be the most negative correlation, whereas *PINK1* were most positively correlated with *ULK1*. Interestingly, *TOMM7* was not correlated with *ULK1*.

### 3.2 GO and KEGG analysis of the DE-MRGs

**Figure 4:**
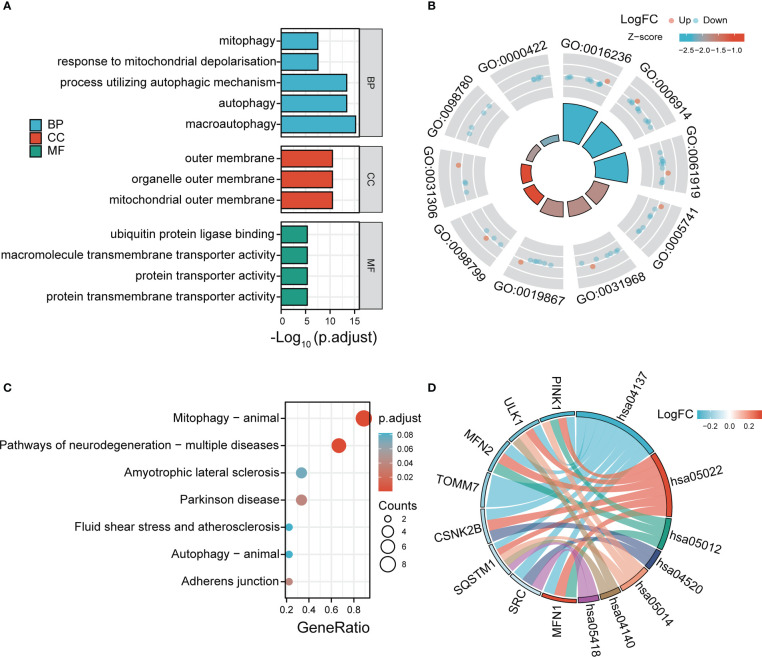
GO enrichment and KEGG analysis of 12 DE-MRGs. **(A)** Bubble plot of enriched GO terms. **(B)** Circos diagram of enriched GO terms. **(C)** Bubble plot of enriched KEGG terms. **(D)** Chord diagram of enriched KEGG terms. BP, biological process; CC, cellular component; MF, molecular function.

According to the GO and KEGG databases, we analyzed the functional enrichment of the DE-MRGs. The results of GO enrichment demonstrated that the most marked terms included macroautophagy, autophagy, process utilizing autophagic mechanism, response to mitochondrial depolarization, mitophagy (biological process); mitochondrial outer membrane (cellular component); protein transmembrane transporter activity and ubiquitin protein ligase binding (molecular function) (**Figures 4A, B**). In KEGG analysis, the DE-MRGs are mainly involved in the process of mitophagy-animal and pathways of neurodegeneration−multiple diseases (**Figures 4C, D**).

### 3.3 Identification of hub genes

**Figure 5:**
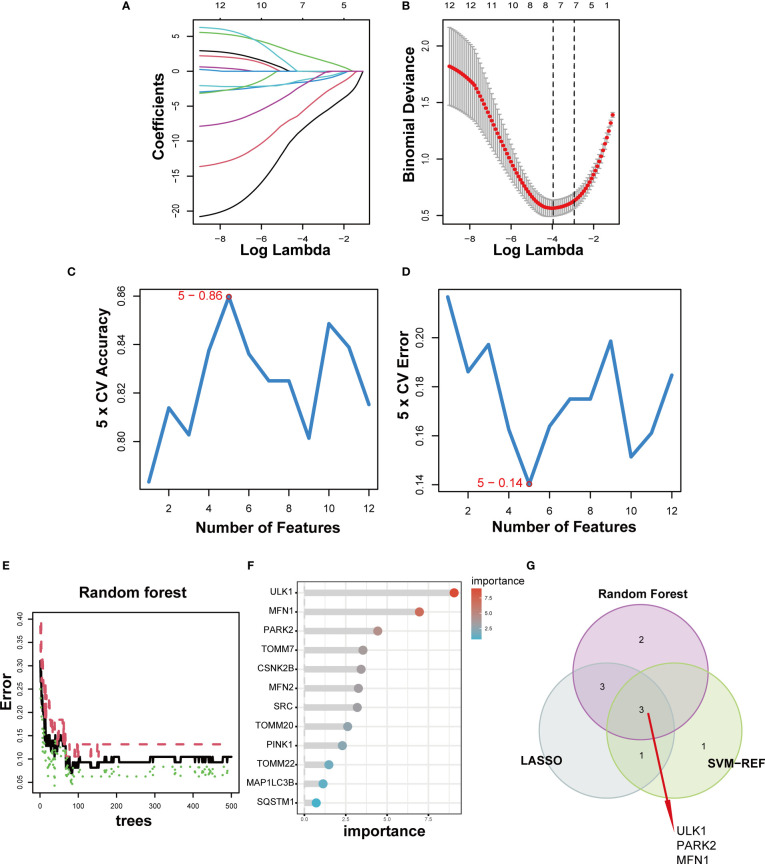
Three DE-MRGs were identified as diagnostic genes for DN. **(A, B)** Regression coefficient path diagram and cross-validation curves in LASSO logistic regression algorithm. **(C, D)** The curve of change in the predicted true and error value of each gene in SVM-RFE algorithm. **(E, F)** The identification of feature importance based on random forests. **(G)** Venn diagram demonstrates the intersection of diagnostic markers obtained from the three algorithms.

For a better understanding of the diagnostic potential of DE-MRGs, we then constructed a prediction model for the diagnosis of DN using three different algorithms to distinguish the DN patients from healthy controls. Seven out of twelve DN-related features of non-zero coefficients were screened using the LASSO algorithm (**Figures 5A, B**). Next, features were selected and five genes were identified as the best candidates for DN based on SVM-RFE (**Figures 5C, D**). Then we identified feature importance using random forests and the top eight genes were selected as diagnostic genes (**Figures 5E, F**). Finally, we intersect the candidate genes acquired from the SVM-RFE, LASSO, and RF models, and 3 hub genes (*ULK1*, *PARK2* and *MFN1*) were recognized for follow-up steps (**Figure 5G**).

### 3.4 Performance of hub genes to diagnose diabetic nephropathy in the training set

**Figure 6:**
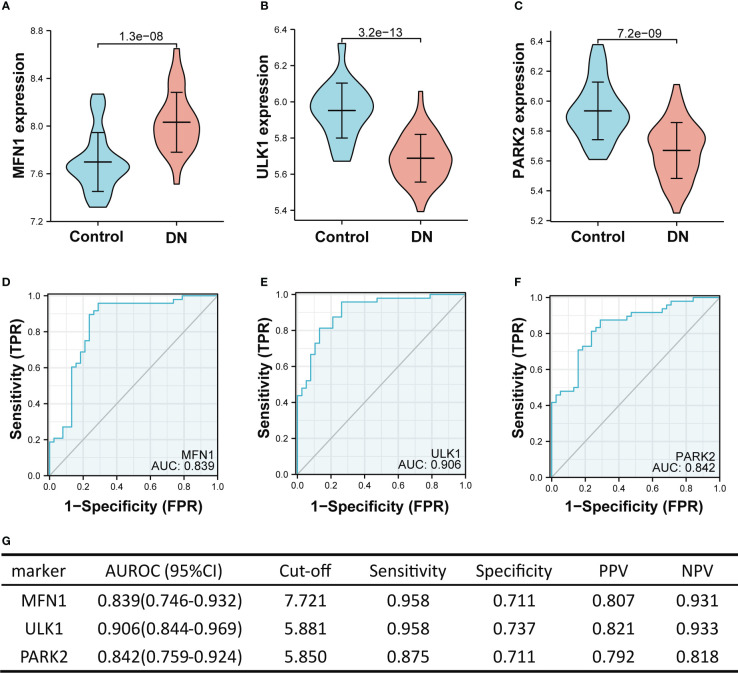
The performance of the three hub genes to diagnose DN in the training set. **(A–C)** Expression difference of the three hub genes in DN and control groups. **(D–F)** The ROC curve of the three hub genes in DN and control groups. **(G)** Diagnostic value of the three hub genes for differentiating DN from control groups. PPV, positive predictive value; NPV, negative predictive value; AUROC, area under the receiver operating characteristics curve; CI, confidence interval. Data were analyzed by Wilcoxon tests or Student’s *t*-tests.

In the training set (GSE104948 and GSE96804), MFN1 was significantly overexpressed in DN compared with the control, while the expression of *ULK1* and *PARK2* were reduced in the DN (all *p* < 0.001, **Figures 6A–C**). The ROC curve showed the AUROC of *MFN1* was 0.839 (95% CI 0.746–0.932), with a specificity of 95.8% and a sensitivity of 71.1% (**Figures 6D, G**). For *ULK1*, the AUROC was 0.906 (95% CI 0.844–0.969), and the sensitivity and specificity were 95.8% and 73.7% (**Figures 6E, G**). The sensitivity, specificity, and the AUROC of *PARK2* were 87.5%, 71.1%, and 0.842, respectively (**Figures 6F, G**).

### 3.5 Performance of hub genes to diagnose diabetic nephropathy in the validation set

**Figure 7:**
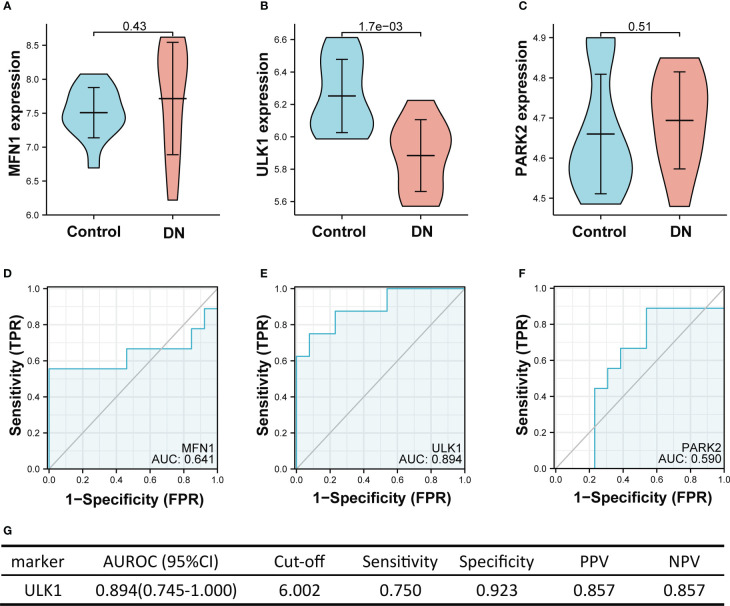
The performance of the three hub genes to diagnose DN in the validation set. **(A-C)** Expression difference of the three hub genes between DN and control groups. **(D–F)** The ROC curve of the three hub genes in DN and control groups. **(G)** Diagnostic value of *ULK1* for differentiating DN from control groups. PPV, positive predictive value; NPV, negative predictive value; AUROC, area under the receiver operating characteristics curve; CI, confidence interval. Data were analyzed by Wilcoxon tests or Student’s *t*-tests.

**Figure 7** showed the value of three mitophagy-related hub genes in the diagnosis of DN in the validation set (GSE30528). The expression of *ULK1* was significantly lower in DN groups than in controls (mean 6.252 vs. 5.884, *p* < 0.01), whereas there was no significant difference in expression of *MFN1* (*p* = 0.43) and *PARK2* (*p* = 0.51) between DN and control. The ROC curve indicated that *ULK1* had excellent performance in diagnosing DN, with AUROC of 0.894 (**Figure 7E**). Interestingly, these results are in conformity with the differential expression of tubulointerstitium samples between DN and controls, with only *ULK1* displaying difference in expression (**Figure 2E**).

### 3.6 Immune infiltration analysis

**Figure 8:**
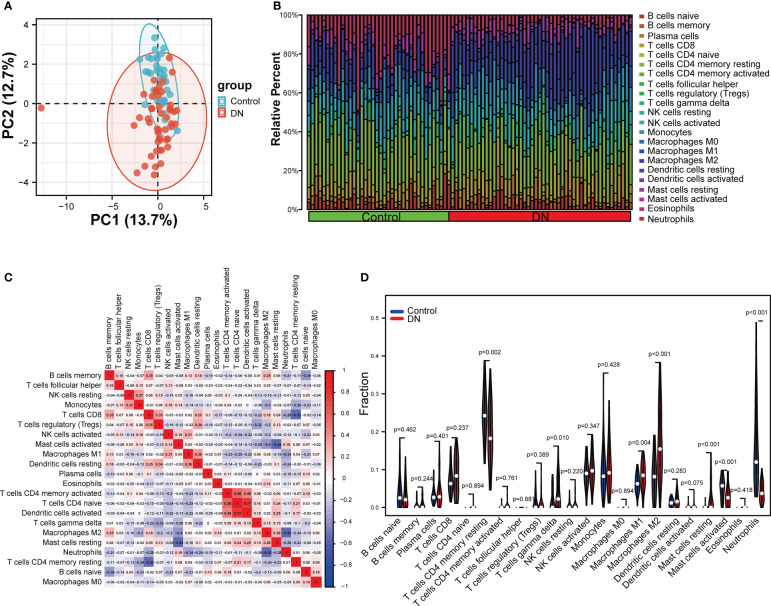
Evaluation and visualization of immune cell infiltration. **(A)** The PCA plot showing immune cell infiltration between DN and control samples. **(B)** Stacked histogram comparing DN and control samples for the immune cell percentage. **(C)** Correlation heat map of 22 types of immune cells. **(D)** Violin diagram showing 22 types of immune cells in proportion. Data were analyzed by Wilcoxon tests.

To explore whether the expression levels of mitophagy-related genes were related to immunity, immune infiltration of DN was assessed using the CIBERSORT algorithms. The analysis of PCA clusters showed that there was a huge distinction between the DN and control samples for immune cell infiltration (**Figure 8A**). Using the par function, the immune cell percentage was calculated and the stacked histogram was plotted (**Figure 8B**). Correlation heatmap drawn to assess the correlation among 22 immune cell infiltrations showed that activated dendritic cells, native CD4 T cells, and CD4 memory T cells had a significant positive relation. Moreover, native CD4 T cells and activated dendritic cells also had a positive relation. Resting mast cells had a significant negative correlation with activated mast cells. A negative correlation was also observed between CD8 T cells and resting CD4 memory T cells, and also between neutrophils and M2 macrophages, respectively (**Figure 8C**). As seen in the violin plot of the difference among 22 immune cell infiltrations (**Figure 8D**), gamma delta T cells, M1 macrophages, M2 macrophages and resting mast cells in DN had a high infiltration compared with control sample, while resting CD4 memory T cells, activated mast cells and neutrophils were less infiltrated.

### 3.7 Correlation analysis between *ULK1* and infiltrating immune cells

**Figure 9:**
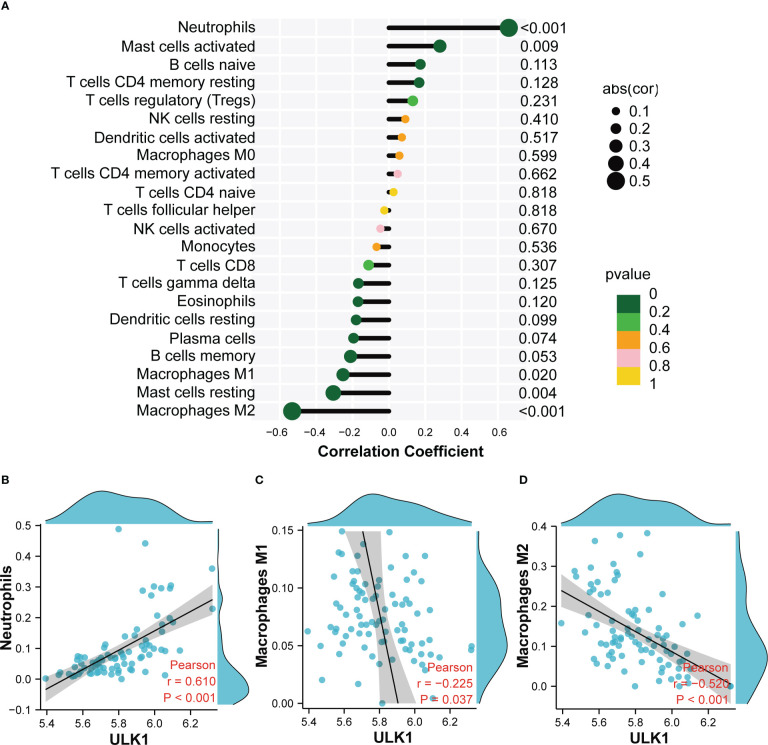
Correlation analysis between *ULK1* and infiltrating immune cells. **(A)** Correlation Analysis diagram showing the correlation between *ULK1* and Infiltrating Immune Cells. **(B)** Scatter diagram indicating the correlation between *ULK1* expression and Neutrophils. **(C)** Scatter diagram indicating the correlation between *ULK1* expression and Macrophages M1. **(D)** Scatter diagram indicating the correlation between *ULK1* expression and Macrophages M2. Correlation analysis was assessed using Pearson correlation.

Based on the immune cell infiltrates, the expression of *ULK1* was found to be positively related to neutrophils (*r* = 0.61), whereas negatively related to M1 macrophages (*r* = −0.225) and M2 macrophages (*r* = −0.52) (**Figure 9**).

### 3.8 Clinical correlation of *ULK1* with renal function

**Figure 10:**
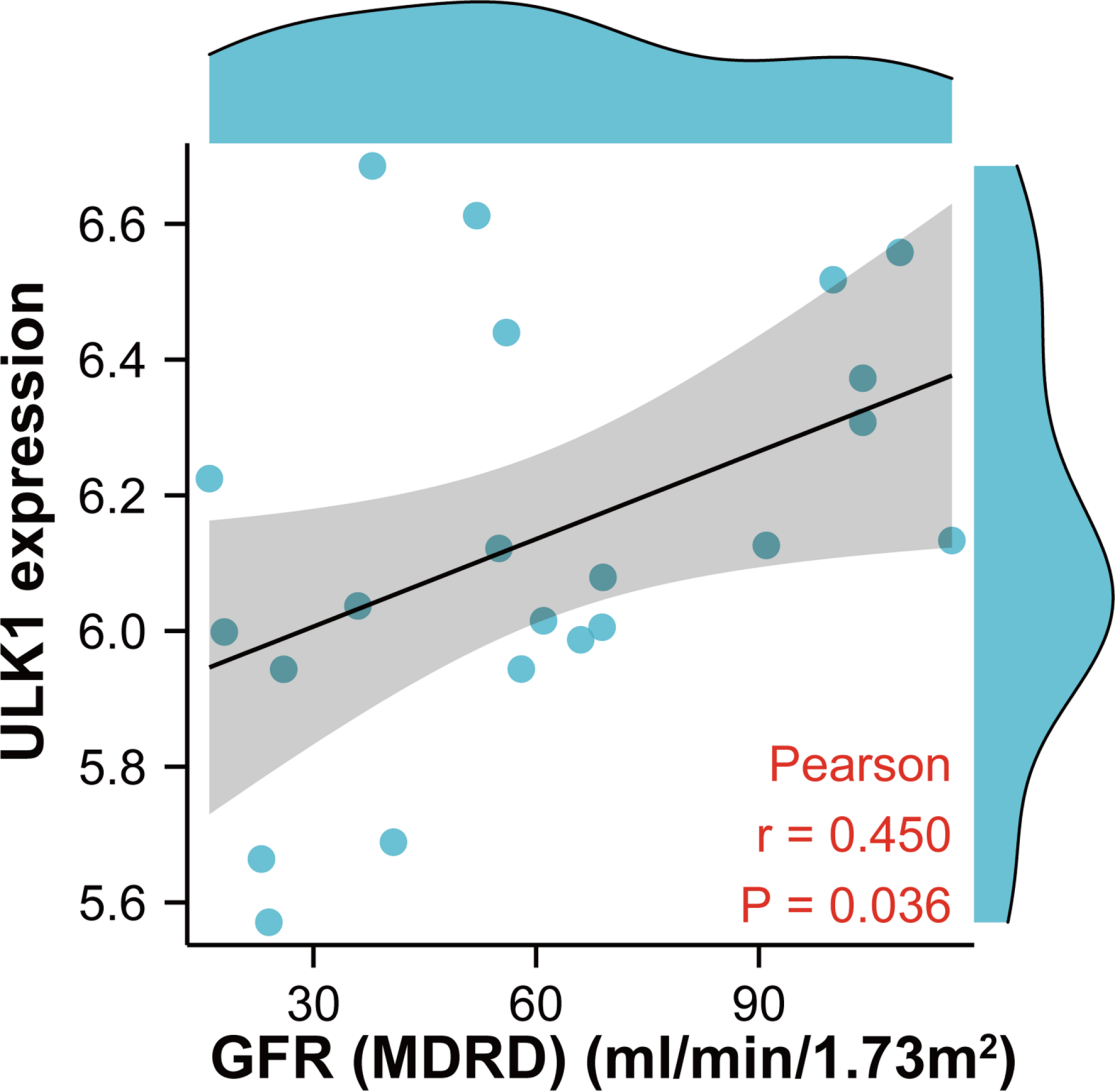
Scatter diagram indicating the relationship between *ULK1* expression and renal function (glomerular filtration rate). Correlation analysis was assessed using Pearson correlation.

To further confirm the role of MRGs in DN, correlation analysis between *ULK1* and GFR was performed with the Nephroseq database (**Figure 10**). *ULK1* was positively related to GFR, revealing that *ULK1* may exert a protective effect against DN.

## 4 Discussion

Diabetic nephropathy is a severe clinical syndrome but lacks specific clinical manifestations. Current diagnostic criteria for DN includes serum creatinine levels, estimated GFR, microalbuminuria, and urinary microalbumin to creatinine ratio. Despite this, these methods are not yet capable of delivering comprehensive diagnoses. Thus, it is essential to explore potential biomarkers with high specificity and sensitivity of DN from multiple perspectives.

Numerous studies suggested that dysregulation of mitophagy promotes the progression of DN. Disorders of mitophagy affect the morphology and function of multiple cells in renal tissue, resulting in decreased glomerular filtration rate, increased proteinuria, glomerulosclerosis, and renal interstitial fibrosis (35, 36). Several cancer-related studies had been performed to explore mitophagy-related genes. For instance, Zhuo et al. constructed a signature of MRGs as a new prognostic model to predict the survival of pancreatic cancer patients (37). Another recent study analyzed the prognostic value and clinical significance of MRGs in hepatocellular carcinoma (38). However, the bioinformatic analysis of MRGs in DN, to our knowledge, has not yet been explored until now.

In this study, we firstly identified 12 MRGs in healthy samples and DN samples through GEO database analysis. GO and KEGG analysis demonstrated that there were several enrichment terms related to autophagy and mitophagy, suggesting that 12 MRGs are actively involved in the process of mitophagy and may be critical for the development of DN. To screen potential diagnostic biomarkers for DN, we performed three different algorithms (SVM-RFE, LASSO and RF) on the above 12 DEGs and identified three genes *ULK1*, *PARK2* and *MFN1* as potential candidate genes. As a key regulator of autophagy, *ULK1* participated in various related metabolic activities of cells. A large body of evidence has indicated that the abnormal expression of *ULK1* is involved in multiple human organ diseases, including neurological diseases, infections, cardiovascular diseases, cancer, and liver diseases (39). *PARK2* encodes Parkin, which is involved in mitochondrial fusion, fission and mitophagy. Parkin is widely expressed in brain, kidney, heart, and other tissues/organs. However, the expression of Parkin is significantly decreased in multiple diabetes-related target organ damages (40). *MFN1* and *MFN2* are the main fusion proteins of mitochondrial outer membrane. Mutations of *MFN1* and *MFN2* are related to the development of neurological diseases, obesity, and vascular diseases (41). In the training sets, the expression of *ULK1* and *PARK2* was markedly down-regulated in DN samples compared with healthy samples, while the expression of *MFN1* was markedly up-regulated. Furthermore, the ROC curves confirmed their robust ability to screen DN samples from normal samples. However, the analysis of the validation set demonstrated that only *ULK1* but not *PARK2* or *MFN1* had a significant difference in the expression between healthy and DN samples, indicating that *ULK1* may be a reliable biomarker for DN.

As the main regulator of initiating general autophagy and mitophagy, ULK1 is strictly regulated by AMPK and MTOR, which are key energy-sensing kinases. AMPK activates ULK1 by phosphorylation of Ser317 and Ser777, while MTOR prevents ULK1 activation by phosphorylating ULK1 in Ser757 (42). The AMPK/ULK1 axis plays a vital role in promoting mitophagy (43, 44). Moreover, ULK1 can directly phosphorylate mitophagy receptors such as FUNDC1, BNIP3, NIX, BCL2L13, and VCP/p97 (45–47). Previous study shows that the mitochondrial outer membrane E3 ligase MUL1 can ubiquitinate ULK1 and regulate selenite-induced mitophagy (48). As a serine/threonine kinase, ULK1 not only participates in the initiation of autophagy (49), but also regulates the maturation of autophagosomes (50) by promoting both ATG5-ATG7-dependent and -independent autophagy pathways (51). Impaired autophagy is involved in the pathophysiology of DN, which increases ROS formation, induces renal cell damage and apoptosis, and mediates inflammatory responses and fibrosis (52). In this study, we found that the expression of *ULK1* in both glomeruli and tubulointerstitium tissues was significantly down-regulated, and further demonstrated that *ULK1* has excellent diagnostic performances (AUC > 0.89). The expression of *ULK1* has been previously reported to be significantly decreased in the kidneys of DN patients (53), and in our study, it was positively related to GFR in patients with DN. This is consistent with the view that mitophagy plays a protective role in DN kidneys (15, 54, 55).

Mitophagy restricts the secretion of inflammatory cytokines and directly regulates mitochondrial antigen presentation and immune cell homeostasis (56, 57). Furthermore, mitophagy can protect cells from inflammation by regulating the adaptive immune response of dendritic cell-T cell synapses, CD8 T cells, and memory NK cells (58, 59). The results of immune infiltration analysis indicated that the expression of *ULK1* was positively related to neutrophils and activated mast cells, whereas it was negatively related to M2 macrophages, M1 macrophages and resting mast cells. It has been reported that mast cells were involved in renal interstitial fibrosis and were closely associated with serum creatinine level in DN (60). Deposition of macrophages has been found in the kidney tissue of DN, indicating decreased renal function in DN patients (61). It is generally accepted that M1 macrophages are mainly pro-inflammatory while M2 macrophages are mainly anti-inflammatory (62). In fact, a previous study showed that both M1 and M2 macrophages had higher infiltration in diabetic nephropathy subjects than healthy subjects (63), which is consistent with our results. As DN progresses, macrophages may undergo a phenotype shift with a change from a classically activated M1 to an alternatively activated M2. Furthermore, studies have also showed that mitophagy can reduce inflammation and fibrosis in DN by regulating the phenotype of M1/M2 macrophages (64). In summary, infiltrating immune cells participate in the occurrence and development of DN, and the abnormal immune status may be improved by targeting *ULK1* in the future.

In our study, the MRGs were obtained from the Reactome database, which is an emerging database and has been widely used in several studies (65–69). However, it did not include some mitophagy receptors such as BNIP3, p62, OPTN and so on. Thus, merging the Reactome database and other database such as KEGG together, might be a better method to obtain more comprehensive MRGs in the future study. Additionally, the lack of experimental validation of the samples is a limitation of our study. The sample numbers from two datasets in this study is limited and it is necessary to select more datasets and confirm our results in a larger DN cohort.

## 5 Conclusions

Our study indicates that the mitophagy-related gene *ULK1* may be a valuable biomarker in the diagnosis of DN. We also demonstrate the potential association of *ULK1* and infiltrating immune cells, suggesting its important role in the development of DN, thereby providing a new insight into the prevention and treatment of DN.

## Data availability statement

The original contributions presented in the study are included in the article/Supplementary Material. Further inquiries can be directed to the corresponding authors.

## Ethics statement

Ethical review and approval was not required for the study involving human participants data from other published articles or databases, in accordance with the local legislation and institutional requirements.

## Author contributions

PW and Z-SL concepted and designed the experiments, revised and approved the final manuscript. Y-YY performed the experiments and collected the data, analyzed and interpreted the data, and draft the manuscript. Z-XG, Z-HM and D-WL provided critical comments and revised the manuscript. All authors contributed to the article and approved the submitted version.
